# Hydrodynamic Tweezers: Trapping and Transportation in Microscale Using Vortex Induced by Oscillation of a Single Piezoelectric Actuator

**DOI:** 10.3390/s18072002

**Published:** 2018-06-22

**Authors:** Xiaoming Liu, Qing Shi, Yuqing Lin, Masaru Kojima, Yasushi Mae, Qiang Huang, Toshio Fukuda, Tatsuo Arai

**Affiliations:** 1 School of Mechatronical Engineering, Beijing Institute of Technology, Beijing 100081, China; liuxiaoming555@bit.edu.cn (X.L.); yuqing@bit.edu.cn (Y.L.); qhuang@bit.edu.cn (Q.H.); tofukuda@nifty.com (T.F.); tarai118@jcom.zaq.ne.jp (T.A.); 2 Department of Systems Innovation, Osaka University, Osaka 560-8531, Japan; kojima@arai-lab.sys.es.osaka-u.ac.jp (M.K.); mae@sys.es.osaka-u.ac.jp (Y.M.)

**Keywords:** micromanipulation, hydrodynamic force, noncontact manipulation, trapping, transportation

## Abstract

The demand for a harmless noncontact trapping and transportation method in manipulation and measurement of biological micro objects waits to be met. In this paper, a novel micromanipulation method named “Hydrodynamic Tweezers” using the vortex induced by oscillating a single piezoelectric actuator is introduced. The piezoelectric actuator is set between a micropipette and a copper beam. Oscillating the actuator at a certain frequency causes the resonance of the copper beam and extend 1D straight oscillation of the piezoelectric actuator to 2D circular oscillation of the micropipette, which induces a micro vortex after putting the micropipette into fluid. The induced vortex featuring low pressure in its core area can trap the object nearby. A robotic micromanipulator is utilized to transport the trapped objects together with the micropipette. Experiments of trapping and transportation microbeads are carried out to characterize the key parameters. The results show that the trapping force can be controlled by adjusting peak-peak voltage of the sinusoidal voltage input into the piezoelectric actuator.

## 1. Introduction

Trapping and transporting micro objects are essential processes in cell injection, cell measurement, cell surgery, bottom-up cell assembly, microdevice tests and assembly of electronic/photonic micro components [1,2,3,4,5,6]. Generally, the existing trapping and transportation methods in micro scale can be divided into two types: contact and noncontact micromanipulations [7].

In contact micromanipulation, robotic micromanipulators have been a fundamental tool for all this time. They can drive the end-effector mounted on the micromanipulators to grasp and further transport objects by the precise physical contact [8,9,10]. This method provides efficient automatic operations and flexible operations of objects with multiple sizes/shapes. However, the dominant adhesion force in microscale causes the release process difficult, and the mechanical contact has potential harm to the cells.

Noncontact trapping and transportation driven by field force in fluidic medium avoid the problems in contact manipulation, so it has been widely applied in biological and medical fields in which the requirement of the cell viability is strict. However, field forces capable of noncontact micromanipulation are limited. Optical tweezers, the most widely used tool in single cell manipulation, generate attractive force based on the strong electric field gradient in the narrowest point of the highly focused laser beam [11,12,13]. Trapping by dielectrophoretic (DEP) is based on the force acting on the polarized object by the non-uniform electric field [14,15]. Acoustic tweezers can trap and move the object by the force generated by the acoustically generated flow [16,17,18,19]. However, effects of the optical tweezers, DEP and acoustic tweezer on the cell viability are still debated, and the system setups for generating the three field forces are extremely complex and costly. Moreover, the three field forces highly depend on the medium and object properties, which makes the force control complicated. In the past two decades, microfluidic chips using the hydrodynamic force generated by the designed geometry have attracted lots of researchers in the biological field, since it guarantees the cell health in the operation [20,21,22]. However, the predesigned geometry inside the microfluidic chips limits the flexible operations of objects with different shapes/sizes, and the closed environments make it difficult to collect the operated objects or access other tools. Thus, different methods have been developed to generate flow for noncontact micromanipulation. Micro-robots driven by magnetic field were utilized to generate flow for noncontact trapping and transportation in microscale [7,23]. Flow around the optically controlled microbubble was applied to assembly in microscale [5]. Concept of open chip based on vortex generated by vibrating pillar array realized the egg cell transportation and extraction [3]. These methods show the great potential of the hydrodynamic force in noninvasive biological micromanipulation. 

In this paper, we describe a simple micromanipulation method using the vortex induced by oscillating a micropipette circularly. This method has the merits of both the contact and noncontact micromanipulations. A piezoelectric actuator is included in a common robotic micromanipulator with a motorized stage and a micropipette as the end-effector. The piezoelectric actuator is used to achieve circular oscillation of the micropipette which can induce a vortex in the fluid medium. The vortex features decreasing pressure from the distance to the core region. The pressure difference can form the hydrodynamic force for trapping the micro object close to the micropipette, and the trapped object can be transported together with the micropipette without any physical contact. It is proved bio-friendly to living cells and capable of the efficient parallel operation with multiple objects. Besides, the trapping force can be easily controlled by the oscillation amplitude of the piezoelectric actuator, and the system is simple and costless.

## 2. Concept of Hydrodynamic Tweezers

**Figure 1:**
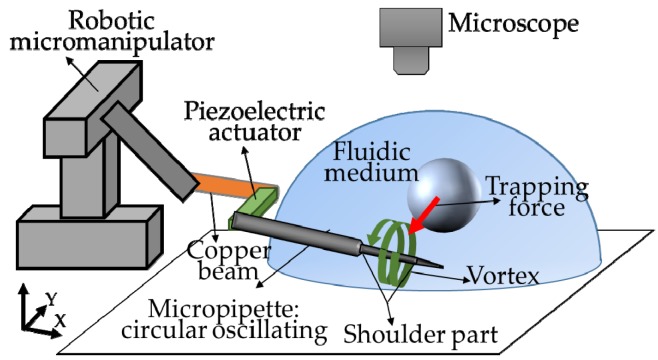
Concept of trapping and transportation using vortex induced by oscillation of a single piezoelectric actuator.

This method is inspired by the phenomenon found in the experiments of active release by oscillating the piezoelectric actuator, which is the released object can be trapped again by continuously vibrating the piezoelectric actuator [10]. As shown in Figure 1, we modify a common micromanipulator system through setting a piezoelectric actuator between a copper beam and the micropipette. The copper beam, piezoelectric actuator and micropipette are positioned together by a robotic micromanipulator. By oscillating the piezoelectric actuator at a certain frequency, the micropipette mounted on it can oscillate circularly, which is able to induce a vortex around the micropipette in microscale. The vortex featuring low pressure in the core region can be applied to trap the micro objects nearby and transport them together with the micropipette. In this section, we will analyze this method from two aspects: the generation of micropipette’s circular oscillation and induced vortex.

## 3. Design and Analysis

### 3.1. Circular Oscillation by Single Piezoelectric Actuator

**Figure 2:**
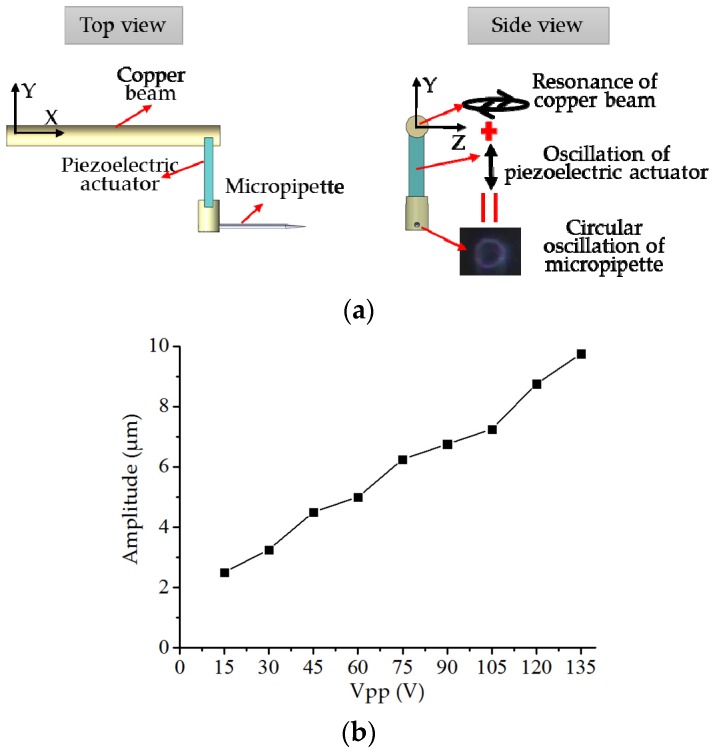
Circular oscillation of the micropipette using the resonance of the copper beam: (**a**) mechanism of generating circular oscillation of the micropipette and (**b**) relationship between Vpp of the sinusoidal voltage input into the piezoelectric actuator and the amplitude of the circular oscillation of the micropipette.

To achieve circular oscillation of the micropipette, a cantilever structure is designed. As shown in the top view in Figure 2a, the structure includes a beam, a piezoelectric actuator, and a micropipette. Oscillating the piezoelectric around the resonance frequency of the cantilever can cause obvious oscillation of the beam. The beam is made of copper to achieve a low resonance frequency. As shown in the side view in Figure 2a, the displacement of the micropipette is the vector sum of the copper beam’s resonance and the piezoelectric actuator’s oscillation. The oscillation of piezoelectric actuator is along the Y axis. The copper beam’s resonance is mainly in Y-Z plane, and displacement in X direction caused by the swing of the cantilever can be ignored. By adjusting the piezoelectric actuator’s oscillating frequency around the cantilever structure’s resonance frequency, we can change the distribution of copper beam’s resonance on the Y and Z axis. Based on this, as the vector sum, the circular oscillation of the micropipette could be achieved. In our experimental setup, at frequency of 350 Hz, circular oscillation can be achieved.

By adjusting Vpp of the sinusoidal voltage input into the piezoelectric actuator, amplitude of the piezoelectric actuator’s oscillation and the resonance amplitude of the copper beam can be changed equidistantly. It provides a way to control the amplitude of the circular oscillation the Figure 2b clearly shows the relationship between Vpp of the sinusoidal voltage input into the piezoelectric actuator and the amplitude of the circular oscillation of the micropipette.

### 3.2. Induced Vortex Analysis by Simulation

To analyze the vortex induced by the circular oscillation of the micropipette, we set up a rotatory 2D model in Comsol Multiphysics. Navier-Stokes equation rules the momentum balance, and the continuity equation rules the mass conservation. Water is selected as the fluidic medium, rotation velocity is set to 350 rps and the area of the fluidic environment is set to 300 μm × 300 μm. 

**Figure 3:**
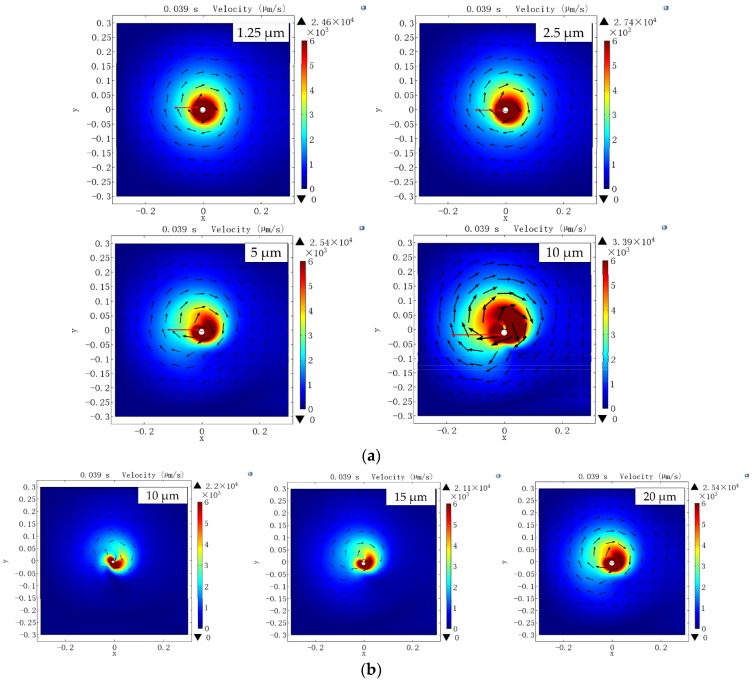
Simulation results of the vortex induced by circular oscillation of the micropipette: (**a**) flow velocity distribution with different circular oscillation amplitudes and (**b**) flow distribution with different outer diameters of the micropipette.

At first, the outer diameter of the micropipette is set to 20 μm, and circular oscillation amplitude changes from 1.25 μm to 10 μm. As the simulation results shown in Figure 3a, the flow velocity distribution clearly shows the induced vortex, and the flow velocity decreases from the core area to the edge. Besides, comparing the circular oscillation with amplitudes of 1.25 μm, 2.5 μm, 5 μm, and 10 μm, we find the area of the flow field highly depends on the circular oscillation amplitude which could influence the maximum distance between the vortex center and the object that can be trapped (trapping range). The flow velocity is also influenced by the circular oscillation amplitude. We can see clearly the red area of the 10 μm amplitude (flow with high velocity) is much bigger than the other amplitudes, which could influence the trapping force during the transportation. To achieve a high transportation velocity, strong trapping force is necessary.

Then, we set the amplitude of the micropipette’s circular oscillation to 10 μm, and change the outer diameter of the micropipette. As shown in Figure 3b, the outer diameter also has a significant effect on the flow velocity in both the flow field area and the flow velocity. The bigger outer diameter the micropipette has, the bigger flow field area and the higher flow velocity can be achieved. It indicates that the outer diameter can also influence the trapping range and the maximum transportation velocity.

In this section, by simulation of the vortex induced by the circular oscillation of the micropipette, the outer diameter of the micropipette and the amplitude of the circular oscillation have significant impact on the flow field area and the flow velocity which will further influence the trapping range and trapping force.

### 3.3. Trapping and Transportation by Vortex

**Figure 4:**
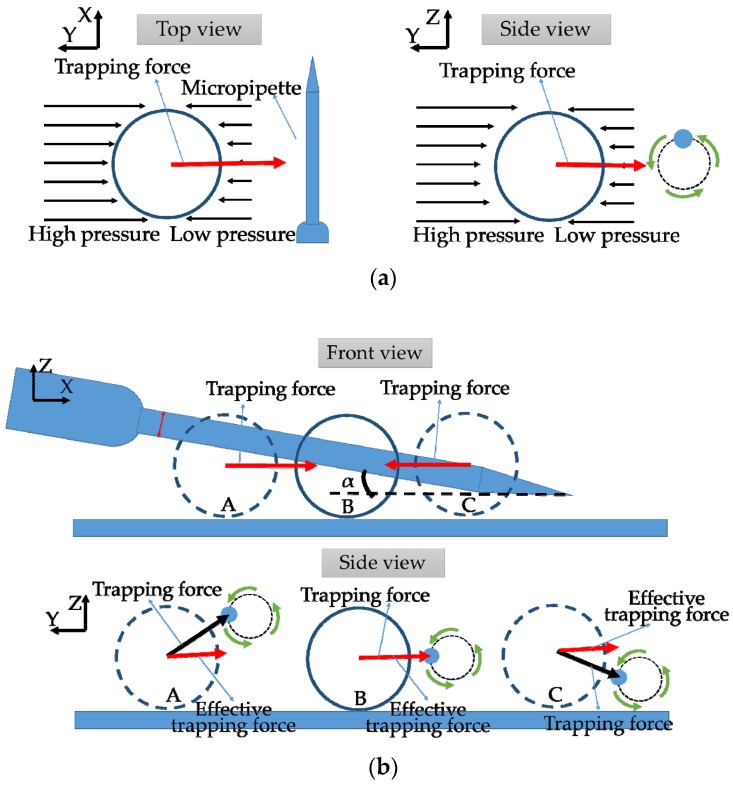
Mechanism of the trapping and transportation: (**a**) along the Y axis; and (**b**) along the X axis.

The trapping mechanism of the proposed method is mainly based on one feature of the micropipette that is the decreasing pressure from the distance to the core region of the vortex. As shown in the Figure 4a, the difference of the pressures exerting on the side near the vortex center and the side far can generate a trapping force to force the object in the flow field to move close to the micro pipette and stay close during the transportation along the Y axis. The bottom under the micro object or the gravity will stop the trapped object to rotate around the micropipette.

Mechanism of transportation on the X axis (along the micropipette) is shown in Figure 4b. In the proposed trapping and transportation method, we set the micropipette on the bottom with a small angle between the micropipette and the bottom (α). If the micro object stays at the B position, the center of the object and the corresponding part of the micropipette are in a same horizontal plane. In this case, the trapping force is horizontal. However, when the object stays at A and C positions, the trapping force has components in both the horizontal direction and the straight direction. The object with a density bigger than water always lies or moves on the bottom in the horizontal plane. Thus, the component of the trapping force in straight direction is offset by the gravity of the support force. The effective trapping force (horizontal component of the trapping force) acting on the object in A and C is smaller than B position. The object tends to go to the position which has the maximum trapping force. It means the object will move to the position where its center and the micropipette are in the same horizontal plane. The angle α could influence the effective trapping force gradient along the X axis, which could affect the transportation velocity along the X axis.

## 4. Experimental Setup

**Figure 5:**
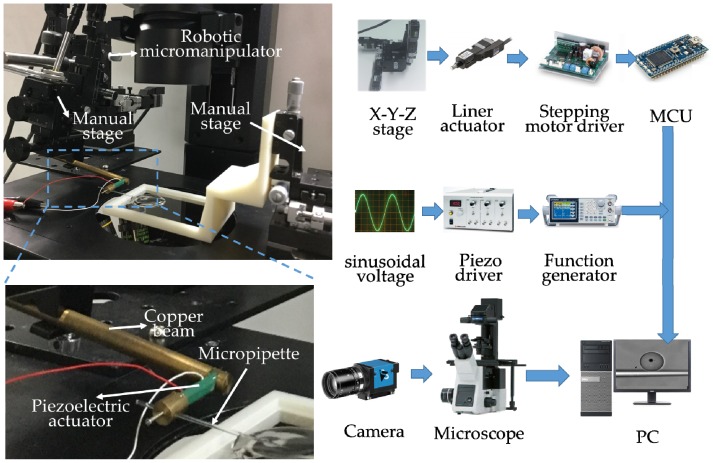
Experimental system setup.

To demonstrate the performance of the proposed method and characterize the key parameters, we have set up a robotic micromanipulation system mainly including the robotic micromanipulator controlling, piezoelectric power supplying and microscope imaging. The robotic micromanipulator was assembled by a 3 DOF stage (TAM-655, Sigmakoki, Japan) and three stepping motors (SGSP-13ACT-B0, Sigmakoki, Japan) powered by drivers (SG-55MA, Sigmakoki, Japan). In piezoelectric power supplying system, the sinusoidal voltage was first generated by the function generator (AFG-2225, GW Instek, China), and then was amplified by the piezo driver (PZJ-0.15P, Matsusada Precision Inc., Japan). The inverted microscope (IX 73, Olympus, Japan) connecting with a digital camera (640 × 480, Imagingsource, Germany) provides the image feedback. With the setup shown in Figure 5, the frequency that could generate the circular oscillation of the micropipette is 350 Hz.

## 5. Experiments and Results

In this part, microbeads (7602 A, Duke Scientific, USA) with the outer diameter of 97 μm and the density of 1.05 g/cm^3^ were used to demonstrate the claimed trapping and transportation method and characterize the key parameters that could influence the trapping or transportation.

**Figure 6:**
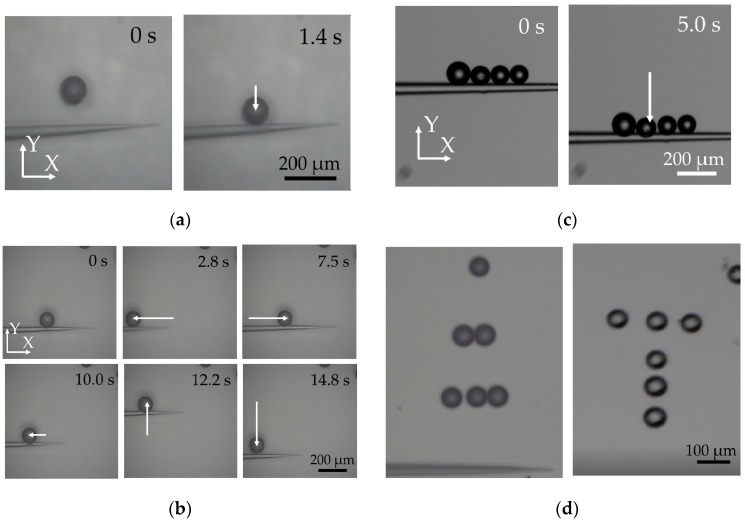
Experimental results of trapping and transportation of the micro beads with an outer diameter of 97 μm: (**a**) trapping of single microbead; (**b**) single microbead transportation in the X-Y plane; (**c**) multiple object transportation; and (**d**) triangle array and “T” array assembled by microbeads.

The micropipette with a shoulder part (outer diameter of 20 μm) was used and put into the water with 50 μm distance between the middle of the shoulder part and the bottom. α was set to 10°. Vpp of the input sinusoidal voltage was 135 V. As shown in Figure 6a, the microbead which stays in 200 μm trapping range was successfully trapped within 1.4 s. The trapping process has an obvious acceleration. Then, the trapped microbead was transported together with the micropipette (Figure 6b) to the left of visual field, then from the left to the center, upward, and downward finally. The whole process took 14.8 s. Besides, four microbeads were transported together up and down to demonstrate the method’s ability of parallel operation of multiple objects. Finally, with the proposed method, a triangle array assembled by 10 microbeads and a “T” array including 6 microbeads were achieved.

From the simulation of the vortex induced by the circular oscillation of the micro pipette, the outer diameter of the micropipette and Vpp of the sinusoidal voltage input into the piezoelectric actuator were found to have a significant influence on the flow field area and flow velocity. In this part, these two parameters and the angle between the micropipette and the bottom (α) have been characterized by experiments with microbeads.

**Figure 7:**
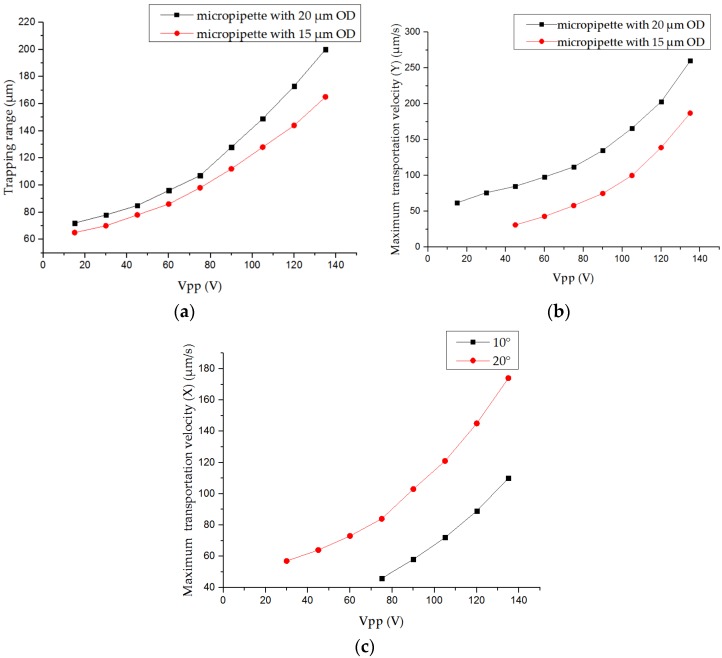
Experimental results of key parameter characterization with microbeads: (**a**) influence of the outer diameter of the micropipette’s shoulder part and Vpp of the input sinusoidal voltage on the trapping range; (**b**) influence of the outer diameter of the micropipette’s shoulder part and Vpp of the input sinusoidal voltage on the maximum transportation velocity along the Y axis; and (**c**) influence of the angle between the micropipette and the bottom (α) and Vpp of the input sinusoidal voltage on the maximum transportation velocity along the X axis.

The trapping range (the maximum distance between the vortex center and the object that can be trapped) was measured by the micropipette with both 15 μm and 20 μm outer diameters. With each micropipette, Vpp of the sinusoidal voltage input into the piezoelectric actuator was changed from 15 V to 135 V. As the experimental results shown in Figure 7a, they clearly demonstrated the positive correlation between the trapping range and Vpp of the input sinusoidal voltage, and the trapping range using outer diameter of 20 μm was bigger than that of 15 μm. The maximum trapping range we can achieve is 200 μm with 135 V Vpp and 20 μm outer diameter of the micropipette’s shoulder part. The maximum transportation velocity along the Y axis (the transportation velocity means the velocity of pulling objects down using the trapping force) has also been measured after the trapping at each situation. As shown in Figure 7b, the bigger Vpp and bigger outer diameter of the micropipette could provide stronger trapping force to allow higher transportation velocity along the Y axis. The maximum transportation velocity along the Y axis we can achieve is 260 μm/s when the Vpp is 135 V and the outer diameter of the shoulder part of micropipette is 20 μm. To evaluate the effect of the angle between the micropipette and the bottom (α) and Vpp on the maximum transportation velocity, we set α to 10° and 20° and changed Vpp from 15 V to 135 V, respectively. Results shown in the Figure 7c indicated that the maximum transportation velocity along the X axis could be increased by increasing the Vpp of input the sinusoidal voltage or the angle between the micropipette and the bottom. The maximum transportation velocity along the X axis reached 174 μm/s with α of 20° and Vpp of 135 V.

## 6. Discussion

The experimental results first demonstrate the trapping and transportation by applying the proposed methods. Noncontact manipulation of micro objects has been achieved by vortex induced by the circular oscillation of the micropipette. Three key parameters influencing the trapping range and maximum transportation have been characterized by the experiments. However, during the operation, the outer diameter of the micropipette and α cannot be changed easily. Vpp is more suitable for the control of the trapping force. Through changing the Vpp, both big and small trapping range can be reached. Big trapping range can be applied in the efficient multiple object operation, while the small trapping range can be used in the precise single object operation without influencing other uninterested objects. The transportation velocity along the Y axis (pulling velocity by trapping force) reaches 260 μm/s, and velocity along the X axis reaches 110 μm/s. They meet the velocity requirement of most application in micromanipulation. Compared with existing micromanipulation tools, the proposed method is more advanced.

Compared with the contact micromanipulation by robotic micromanipulators, it avoids the difficult release and cell damages caused by the physical contact. Different from the other noncontact micromanipulation methods using the field force including optical tweezers, DEP and acoustic tweezers, the proposed method is based the hydrodynamic field force. Cell health during the operation can be guaranteed. Moreover, it only needs the density of the micro object bigger than the fluidic medium, while the other noncontact methods are very sensitive to the properties of the medium or objects. Compared with the microfluidic chips that use the hydrodynamic force as well, the proposed method generates the hydrodynamic force in an open environment. It has advantages in flexible manipulation of objects with different sizes/shapes, objects collection after operation and access to other tools for complex manipulations. Besides, the time-cost design and fabrication of the microfluidic chips are omitted. Moreover, compared with other noncontact methods that generate flow using micro-robot driven by magnetic field [7,23], flow around optically controlled microbubble [5] and vibrating pillar array [3], the proposed method doesn’t need additional field force or fabrication of the micro-robot/pillar-array. Only a micropipette driven by a piezoelectric actuator is used in the hydrodynamic force generation which is much simpler that other field forces generation. The control of the trapping force can also be easily achieved through changing Vpp of the input sinusoidal voltage.

## 7. Conclusions

A novel noncontact trapping and transportation method in microscale named “Hydrodynamic Tweezers” was introduced. By using the vortex around the micropipette induced by oscillation of a single piezoelectric actuator, objects could be trapped by the low pressure in the core center of the vortex and further transported together with the micropipette by a robotic micromanipulator. Comparing with contact micromanipulation by robotic manipulators and other existing noncontact methods, we can conclude the main advantages of the proposed method are: (1) no damage to the living cell; (2) capable of parallel operation of multiple objects; (3) flexibility of operating objects with different sizes/shapes; (4) controllable trapping force through Vpp of the sinusoidal voltage input into the piezoelectric actuator; and (5) simple and costless system including only a piezoelectric actuator and a common robotic micromanipulator.
